# Search for altered imprinting marks in Mayer–Rokitansky–Küster–Hauser patients

**DOI:** 10.1002/mgg3.426

**Published:** 2018-08-11

**Authors:** Thomas Eggermann, Susanne Ledig, Matthias Begemann, Miriam Elbracht, Ingo Kurth, Peter Wieacker

**Affiliations:** ^1^ Institute of Human Genetics Technical University RWTH Aachen Aachen Germany; ^2^ Institute of Human Genetics University of Münster Münster Germany

**Keywords:** ICR1 hypomethylation, imprinting, Mayer–Rokitansky–Küster–Hauser syndrome, Silver–Russell syndrome

## Abstract

**Background:**

Mayer–Rokitansky–Küster–Hauser syndrome (MRKH) is the second most common cause of primary amenorrhea and characterized by absence of the uterus and the upper part of the vagina. The etiology of MRKH is mainly unknown but a contribution of genomic alterations is probable. A molecular disturbance so far neglected in MRKH research is aberrant methylation at imprinted loci. In fact, MRKH has been reported in patients with the imprinting disorder Silver–Russell syndrome.

**Methods:**

We report on a rare patient with MRKH and SRS due to an ICR1 hypomethylation in 11p15.5. On the basis of this observation we screened a large cohort of MRKH patients (*n* > 100) for aberrant methylation at nine imprinted loci.

**Results:**

We failed to detect any epimutation, thus we conclude that imprinting defects at least at the currently known disease‐relevant imprinted loci do not contribute to the isolated MRKH phenotype. However, it cannot be excluded that altered methylation marks at other loci are involved in the etiology of MRKH.

**Conclusion:**

The molecular basis for MRKH remains unclear in the majority of patients, but future studies on the association between MRKH and ICR1 hypomethylation/SRS will to enlighten the role of epigenetics in the etiology of MRKH.

## 1 INTRODUCTION

Mayer–Rokitansky–Küster–Hauser syndrome (MRKH, Mullerian agenesis, OMIM277000) is the second most common cause of primary amenorrhea (for review: Fontana, Gentilin, Fedele, Gervasini, & Miozzo, 2017). It is characterized by absence of the uterus and the upper part of the vagina, whereas Fallopian tubes and the ovaries are usually of normal morphology and function. Two types of the disease are currently distinguished, MRKH type 1 without further features, and MRKH type 2 with additional malformations especially of the kidneys and the skeleton. Additionally, uterine agenesis and malformations are also associated with other syndromes (for review: Jacquinet, Millar, & Lehman, 2016). The incidence of MRKH has been estimated as 1 in 4,500 to 5,000 females. In the majority of cases, MRKH occurs sporadically, but single families have been reported (Shokeir, 1978). Although a common molecular cause for MRKH is not known, numerous chromosomal copy number variations and gene mutations have been reported (for review: Fontana et al., 2017). Thus, the etiology of MRKH is far from being understood, but it has been postulated that the heterogeneous molecular findings share an involvement in developmental pathways in embryogenesis.

A molecular disturbance which has so far been neglected in the search for the molecular cause of MRKH is aberrant methylation at imprinted loci. In the human genome, approximately 100 genes are genomically imprinted, meaning that they are expressed in a parent‐of‐origin specific manner whereas the expression of the other allele is silenced. One molecular mechanism in imprinting control is DNA methylation, and disturbances of this fine‐tuned methylation and expression of an imprinted gene or region can result in an imprinting disorder (for review: Soellner et al., 2017). Currently, 12 imprinting disorders are known with a broad spectrum of clinical features, including disturbed growth, metabolic disturbances, asymmetry and cognitive disabilities involving different chromosomal loci (chromosomes 6, 7, 11, 14, 15, and 20). In fact, MRKH has been reported in patients with Silver–Russell syndrome (SRS, RSS, OMIM180860), an imprinting disorder associated with a hypomethylation of the imprinting control region 1 (ICR1) in 11p15.5, maternal uniparental disomy of chromosome 7 and other less frequent molecular changes. In addition to severe prenatal and postnatal growth retardation as the key features, SRS is characterized by a relative macrocephaly, asymmetry, a protruding forehead with a characteristic triangular face, and feeding difficulties (for review: Wakeling et al., 2017). The ICR1 hypomethylation is the most frequent finding in SRS, accounting for nearly 40% of patients. SRS belongs to the group of rare diseases, its incidence is estimated to range between 1 in 3,000 to 1 in 100,000 (Price, Stanhope, Garrett, Preece, & Trembath, 1999). In the Estonian population an incidence of 1 in 70,000 has been determined (Vals et al., 2015). MRKH as a comorbidity of SRS has been reported in five cases (Abraham et al., 2015; Bellver‐Pradas et al., 2001; Bliek et al., 2006; Bruce et al., 2010), among them three with ICR1 hypomethylation, and it is therefore recommended to investigate girls with SRS and primary amenorrhoea for MRKH (Wakeling et al., 2017). Interestingly, SRS shows a clinical and molecular overlap with other imprinting disorders (e.g. Temple syndrome, Beckwith–Wiedemann syndrome). Molecular subcohorts of these diseases show a so‐called Multilocus Imprinting Disturbance (MLID; Soellner et al., 2017) which is defined as the aberrant methylation of imprinted loci additional to the disease‐specific differentially methylated region. As the result of the molecular overlap and MLID, similar phenotypes can be observed in different molecular patient cohorts.

Based on the identification of a patient with SRS due to an ICR1 hypomethylation and MRKH, we aimed to identify a link between aberrant methylation at imprinted loci and MRKH. Due to the heterogeneous molecular findings in SRS, we did not restrict our analysis on the ICR1 in 11p15, but included all further clinically relevant imprinted regions in our search. For this purpose, we screened a cohort of more than 100 MRKH patients for epimutations at known disease‐associated imprinted loci. We hypothesized that MRKH might represent one end of the broad phenotypic spectrum of SRS (and other imprinting disorders), and that other features would be only slightly present if at all.

## 2 STUDY COHORT

ICR1 hypomethylation was detected in a 24 years old woman referred for SRS testing due to growth retardation (156 cm, 3rd percentile) and a protruding forehead. Congenital aplasia of uterus and vagina were reported.

The MRKH cohort comprised 53 patients with MRKH type I and 52 patients with a MRKH type II. Patients’ samples have been collected in the context of a study for genetic causes of MRKH. Most of the patients presented in gynecology because of amenorrhea. Ultrasound examination confirmed missing uterus. All patients have a female karyotype 46,XX.

The second cohort consisted of 31 female SRS patients with ICR1 hypomethylation: clinical data were ascertained in the course of an ongoing study on the etiology of SRS, and were documented by a comprehensive clinical questionnaire. Informed consent was obtained from all patients, the study was approved by the ethical committees from the University Hospitals of Aachen (EK‐302‐16) and Münster (2010‐570‐f‐S).

## 3 MATERIALS AND METHODS

To identify altered imprinting marks of differentially methylated regions (DMRs) with a known association with imprinting disorder phenotypes, we used a methylation‐specific multiplex ligation probe‐dependent amplification assay (MS‐MLPA) which targets the DMRs of the genes *PLAGL1* (chromosome 6; transient neonatal diabetes melllitus), *GRB10* and *MEST* (chromosome 7; SRS), *H19* and *KCNQ1OT1* (chromosome 11; SRS, Beckwith–Wiedemann syndrome), *MEG3* (chromosome 14; Temple syndrome, Kagami–Ogata syndrome), *SNRPN* (chromosome 15; Prader–Willi syndrome, Angelman syndrome), *DIRAS* (chromosome 19), and *NESPAS* and *GNAS* (chromosome 20; pseudohypoparathyroidism; Mulchandani–Bhoj–Conlin syndrome). The MS‐MLPA assay (ME034‐A1, MRC Holland, Amsterdam/NL) was applied according to the manufacturers instruction, PCR products were run on an automated sequencer (AB3130, Applied Biosystems, Darmstadt, Germany). In case of the SRS patient with MRKH, two additional MS‐MLPA assays were used (ME030‐C1; ME032‐A1). MLPA raw data were processed with the freely available Coffalyser.Net software (MRC Holland).

## 4 RESULTS AND DISCUSSION

Due to the identification of a SRS patient with MRKH and ICR1 hypomethylation in our SRS cohort and on recent reports on female SRS patients with MRKH features, we analysed a large cohort of MRKH patients for aberrant methylation at imprinted loci. However, we did not identify any epimutation at DMRs on chromosomes 6, 7, 11, 14, 15, and 20. We furthermore searched for MRKH features in a group of SRS patients with ICR1 hypomethylation, but the majority of patients (*n* = 25) was younger than 11 years at the time of referral and thus MRKH might be undiagnosed as only obvious malformations are ascertained by the used questionnaire. Among the six adult ICR1 hypomethylation carriers, the patient reported here was the only showing amenorrhea and MRKH. In fact, SRS is mainly diagnosed in (early) childhood, and systematic follow‐up studies including a documentation of menarche or amenorrhea have not yet been reported. Thus, the number of undetected SRS patients with MRKH might be higher.

Our case is the sixth SRS patient with MRKH in the literature. Abraham et al. (2015) recently summarized five cases, in three of them a ICR1 hypomethylation was identified and thereby the clinical diagnoses of SRS was confirmed. However, the other two patients (Abraham et al., 2015; Bellver‐Pradas et al., 2001) were ascertained on the basis of clinical data suggestive for SRS. In fact, the problem of clinical diagnosis of SRS is the heterogeneity of the disease, and until recently a standardized tool for this purpose was missing. With the recent introduction of the Netchine‐Harbison clinical scoring system (NH‐CSS) for SRS as the standard clinical scoring system (Wakeling et al., 2017), this problem can at least in part be circumvented. The score comprises six clinical parameters which can relatively easily be scored and are mainly based on objective data (being small for gestational age (SGA), postnatal growth retardation (PNGR), relative macrocephaly at birth, protruding/prominent forehead, body asymmetry, and feeding difficulties and/or low body mass index (BMI) in early life). For patients suspicious for SRS but without molecular confirmation, it has been suggested that only patients with a prominent forehead and relative macrocephaly should be diagnosed as clinical SRS. By applying this score in the two patients without molecular confirmation of SRS, it turned out that in the case described by Bellver‐Pradas et al. (2001) the NHS could not be applied because only three of the required scoring items were available. For the patient reported by Abraham et al. (2015), four NHS features were positive and thus the clinical diagnosis of SRS was confirmed.

Despite the uncertainties in clinical diagnosis in one of the six cases with SRS and MRKH features, the data from different cohorts of female SRS patients with an ICR1 hypomethylation indicate that an association between MRKH and SRS is rare So far, MRKH has been reported in three study cohorts: Bliek et al. (2006) reported on one MRKH of seven female ICR1 hypomethylation carriers, Bruce et al. one of 26 patients (of both sexes); in our cohort of 31 female ICR1 hypomethylation carriers, one also suffered from MRKH. In other larger cohorts of SRS patients (Netchine et al., 2007; Wakeling et al., 2017) MRKH was not reported, probably due to the early clinical diagnosis of SRS in these patients.

Considering the incidence of 1:4,000–1:5,000 for MRKH in the general population, the identification of at least four patients with a molecularly confirmed SRS provides evidence for a causal relationship between the two phenotypes. As we hypothesized that MRKH might represent the mild end of the broad phenotypic spectrum of SRS (and other imprinting disorders), we screened a cohort of more than 100 MRKH patients for aberrant methylation at imprinted loci. However, we could not detect any epimutation, thus we conclude that imprinting defects at least at the currently known loci associated with imprinting disorders do not contribute to the isolated MRKH phenotype. In fact, it cannot be excluded that altered methylation marks at other loci are involved in the etiology of MRKH, but there are currently no further evidences for this assumption.

In conclusion, the molecular basis for MRKH remains unclear in the majority of patients, but further studies on the association between MRKH and ICR1 hypomethylation/SRS are required and might help to enlighten the etiology of MRKH.

## CONFLICT OF INTEREST

The authors declare that there is no conflict of interest.
